# Recurrent incarceration of the retroverted gravid uterus at term - two times transvaginal caesarean section: a case report

**DOI:** 10.1186/1752-1947-3-103

**Published:** 2009-11-03

**Authors:** Karin van der Tuuk, Robert A Krenning, Guido Krenning, Wilma M Monincx

**Affiliations:** 1 Department of Obstetrics and Gynaecology, University Medical Center Groningen, University of Groningen, PO Box 30.001, 9700 RB Groningen, The Netherlands; 2 Department of Obstetrics and Gynaecology, Mesos Medical Center Utrecht, Van Heuven Goedhartlaan 1, 3527CE Utrecht, The Netherlands; 3 Department of Pathology and Medical Biology, University Medical Center Groningen, University of Groningen, PO Box 30.001, 9700 RB Groningen, The Netherlands; 4 Department of Obstetrics and Gynaecology, St Antonius Hospital Nieuwegein, PO Box 2500, 3430 EM Nieuwegein, The Netherlands

## Abstract

**Introduction:**

Persistent retroversion of a gravid uterus (incarceration) in the third trimester is an extremely rare diagnosis and is only scarcely been described. Its prevalence may lead to increased foetal mortality and maternal morbidity.

**Case presentation:**

We present a case where a 35-year-old patient had undiagnosed (recurrent) uterine incarceration at term. Operative delivery proved difficult due to distorted anatomy. Therefore, in our case delivery of the fetus through transvaginal caesarean section was required.

**Conclusion:**

This case report discusses the diagnosis and management of (recurrent) incarceration of the retroverted uterus at term resulting in two successful transvaginal caesarean sections. In presenting this case, we aim at improving awareness, diagnosis and treatment of the retroverted incarcerated gravid uterus.

## Introduction

Incarceration of a retroverted gravid uterus has only scarcely been described. The condition is rare and its prevalence may lead to increased fetal mortality and maternal morbidity. Patients with an incarcerated retroverted uterus may present symptoms of urinary retention, lower abdominal pain, constipation or rectal pressure. Diagnosis is difficult due to variable clinical manifestations and operative management is technically complicated because of distorted anatomy.

Here we present a case of (undiagnosed) incarceration occurring in two succeeding pregnancies. In both cases, incarceration resulted in transvaginal caesarean section. In presenting this case, we aim to improve awareness, diagnosis and treatment of the retroverted incarcerated gravid uterus.

## Case presentation

A 35-year-old primigravida, with no significant medical history, presented at 33 weeks gestation because of uncertain presentation of the foetus. Her pregnancy had been asymptomatic. An initial ultrasound reported to show a single foetus in breech position and a complete placenta previa. There were no other signs that made us suspect distorted anatomy. Ultrasound measurements further showed intra-uterine growth restriction (5th percentile) with a normal amniotic fluid index. Doppler examination of the umbilical artery revealed a PI from 1.028 which is within normal range. A primary caesarean section was planned at 38 weeks of gestation.

**Figure 1:**
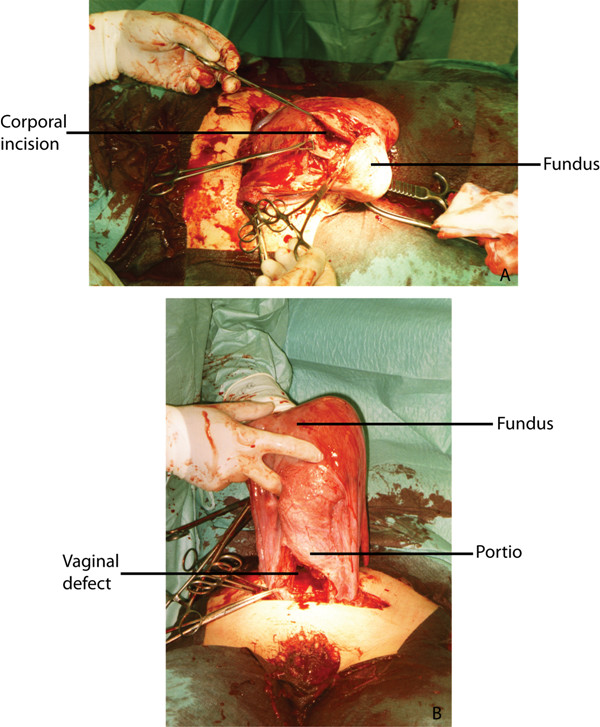
**Photographs taken during SC demonstrating the corporal incision on the posterior side of the uterus (A) and the incision through the anterior and posterior walls of the vagina (B)**.

At 37+6 weeks of gestation, the patient was admitted to the hospital with spontaneous rupture of membranes and loss of amniotic fluid. A caesarean section was performed. During the caesarean section a Pfannenstiel incision was made and the bladder and uterovesical peritoneum were deflected inferiorly. What appeared to be the lower segment was then incised but instead of entering the uterine cavity a second apparent uterine surface was encountered. Although the foetal breech could be felt within the underlying uterine surface, the anatomy was unclear. Therefore, a second transverse incision was made revealing a third uterine surface. After a third transverse incision was made, the uterine cavity was entered, presenting the breech. A live male infant, birth weight 2570 grams (5-10 percentile), was born. Apgar scores were 9 and 10 at 1 and 5 min post partum respectively. Arterial pH was 7.36. After closure of the uterine incision we carefully examined the abdominal organs. The incision appeared to be corporal and on the posterior side of the uterus (Fig. 1A), indicating that the uterus had been retroverted. The fundus was lying in the pouch of Douglas. Hence, the presentation was cephalic (by definition) but had appeared to be breech (by ultrasound).

The placenta previa was misdiagnosed due to distorted anatomy and was really situated in the fundus. No cause for the retroversion was detected. The initial incisions had been made through the anterior and posterior walls of the vault of the vagina, which was stretched by the growing incarcerated uterus. At that point the uterus was almost completely detached (Fig. 1B). The baby was born through the displaced posterior surface incision of the uterine fundus and the vaginal incisions. The uterine blood vessels were intact. After consulting a colleague, it was decided to close both vaginal incisions and restore normal anatomy. The bladder was situated in the normal anatomical position. Due to the uncommon corporal incision, a conservative healing period was chosen and the patient was advised not to become pregnant within the first year. We advised a primary caesarean section at 37 weeks of gestation if a new pregnancy occurred. Postoperative recovery was complicated by an ileus which was treated by nil per os and intravenous hydration. Mother and child left the hospital 11 days after the operation in good condition.

One year later, during her second pregnancy, ultrasound examination again revealed a uterus in retroversion at 18 weeks of gestation. We considered the possibility of turning the uterus to an upward position [1]. However, attempts to correct uterus position later than the 15th week are more likely to fail and may cause complications such as preterm labour [2]. Furthermore, it is recommended by some authors only to intervene if the patient presents with severe symptoms [3,4]. Although we strongly advised the patient to consult us in the early first trimester, we did not see her before 18 weeks of pregnancy and the decision was made not to turn the uterus in an upward position.

The pregnancy progressed asymptomatic and at 37+5 weeks of gestation a repeat transvaginal caesarean section was preformed in the same manner as described above. A girl weighing 2810 grams (10-25 percentile) was born with apgar scores 9 and 9 at 1 and 5 min post partum respectively. The postoperative course was uncomplicated and mother and child left the hospital seven days postoperative. Six weeks after delivery, ultrasound examination showed the uterus in anteverted flexion.

## Discussion

Retroversion of the uterus is found in approximately 11 to 19% of women at the time of conception [5]. Usually the uterus assumes a normal position at about the twelfth week of pregnancy, when the expanding organ enters the abdomen. Retroversion only rarely persists after 14-16 weeks. If retroversion persists, the fundus becomes incarcerated in the pelvic cavity. Incarceration of the gravid uterus occurs in approximately one of 3000 pregnancies [1,2]. Contributing factors can be pelvic adhesions, endometriosis, ovarian tumors, leiomyoma and uterus anomalies [2,4,6-10]. Early recognition of incarceration of a retroverted uterus is of high importance, because correction may still be possible and pregnancy can proceed normally.

The obstetrician should always consider the possibility of turning the uterus to an upward position [1]. In the presented case, we considered turning the uterus in upward position, but no attempts were undertaken due to the late presentation, absence of symptoms and increased risk for premature labour.

Some authors suggest that patients carry more risk of recurrence of retroversion of the uterus [11]. Our case report is the fourth case of recurrent incarceration of the gravid uterus in literature. One previous case, describes a woman with uterus didelphus [8], in the other two cases no plausible cause for the condition were found [9,11]. We recommend that women with a history of an incarcerated retroverted gravid uterus, in later pregnancies should be examined early in pregnancy.

Retroversion of the gravid uterus presenting as late as in the third trimester been scarcely reported. According to Singh and co-workers, only 28 cases were reported in the English literature between 1967 and 2006 [12]. Incarceration may lead to increased foetal mortality and maternal morbidity and diagnosis of uterine incarceration is difficult due to variable clinical manifestations, ultrasound findings and physical examination.

Clinical manifestations occur after the first trimester and can be divided into four categories; [1] Obstetric and gynaecological symptoms, *e.g*. bleeding, miscarriages due to compromised uterine circulation; [2] Pressure symptoms, *e.g*. lower abdominal pain; [3] Urinary symptoms, *e.g*. urinary frequency, dysuria, incontinence; and [4] Gastrointestinal symptoms, *e.g*. rectal pressure, tenesmus, constipation [2,3,10]. Only few cases are described where no obvious causes or symptoms were found [5,7,11], as in our case. Ultrasound examination may show a drawn up bladder along with absence of the anterior uterine wall. However, as in the presented case, ultrasound can be misleading. During the first pregnancy we didn't find signs that made us suspect distorted anatomy. Our patient was diagnosed with a foetus in breech position and a complete placenta previa by ultrasound. However, caesarean section revealed an incarcerated, retroverted uterus; hence the ultrasound based diagnosis was erroneous. Four past cases of uterus incarceration have described similar findings. Similar to the presented case, three cases describe spontaneous rupture of the membranes and oligohydramnios in the setting of placenta previa [4,5,13]. This unusual combination may therefore indicate a uterus incarceration.

Indicators for an incarcerated uterus at physical examination include low fundal height, a filled cul-de-sac and an unreachable cervix 2. The cervix wasn't palpable, but was visible with transvaginal ultrasound, during her first pregnancy the possibility of an incarcerated uterus was not considered. It was unknown whether the patient had a retroverted uterus prior to pregnancy. When uterus incarceration occurs, the cervix becomes displaced upward above the symphysis, making vaginal delivery impracticable. Two previous cases of vaginal deliveries resulted in foetal death [14,15]. Therefore, if incarceration of a retroverted gravid uterus is suspected a caesarean section must be planned. Hence, preoperative recognition of retroversion is essential and can prevent intraoperative complications. Herein, it is of great importance to bear in mind that the distorted anatomy due to uterus incarceration can result in maternal morbidity. Previous cases note trauma to the bladder, vagina, and cervix as well as delivery through the posterior wall of the uterus [1,5,7,12]. The current case illustrates the practical difficulties of this distorted anatomy, which resulted in two transvaginal caesarean sections. Transvaginal caesarean in the setting of retroverted incarcerated uterus has only scarcely been described. Uma and co-workers [10] described a patient with an incarcerated fibroid gravid uterus. They preformed a transvaginal caesarean section resulting in a completely detached uterus at the level of the vagina. Due to this complication, a hysterectomy was performed.

To our knowledge our case is the first case which describes two successful transvaginal caesarean sections. Still, in hindsight we wouldn't recommend a transvaginal caesarean section. Preoperative recognition of retroversion is essential and can prevent intraoperative complications.

Due to the incarceration of the uterus the cervix, vagina and bladder might become elongated. Localization of the elongated vagina, cervix, as well as the urethra and bladder are essential for surgery. Proceeding to caesarean section without correct diagnosis will cause difficulties identifying these structures and in opening the lower uterine segment. This may lead to bladder injuries, vaginal transsection and trans- or supracervical hysterectomy. Magnetic resonance imaging offers a non/invasive method to confirm the diagnosis and to reconstruct the exact anatomy [6]. In this case we unfortunately did not think of this possibility. After this visualization of the anatomy we would recommend a median laparotomy and if possible, restoration of normal anatomy [1,4,12]. When normal anatomy cannot be restored, the incision in the uterus should if possible be made in the lower uterine segment, for this prevents future maternal morbidity. If this is not possible a high (corporal) incision is required.

## Conclusion

In conclusion, the diagnosis of uterus incarceration is rare, rendering case reports the source of most information and improve awareness of this complication. Here, several aspects underscore the need to maintain an index of suspicion for incarceration as findings on ultrasound (*e.g*. previa, breech) are not specific. Late (very late) identification of the incarceration, lack of symptoms, absence of anatomic abnormalities contributing to the incarceration, recurrence of incarceration in the same patient and surgical approach to the caesarean section can be challenging. Because of the increased risk for recurrence in subsequent pregnancies, we stress the importance of pelvic examination before the 15th week of pregnancy. By then, correction of the normal anatomy may still be possible, reducing the risk of foetal and maternal morbidity. When retroversion is suspected, MRI can be used to confirm the diagnosis and to reconstruct the exact anatomy. We don't recommend a transvaginal caesarean section but advise a midline laparotomy.

## Consent

Written informed consent was obtained from the patient for publication of this case report and accompanying images. A copy of the written consent is available for review by the Editor-in-Chief of this journal.

## Competing interests

The authors declare that they have no competing interests.

## Authors' contributions

KT made substantial contributions to conception and design, analysis and interpretation of data, and was the major contributor in writing the manuscript. RAK made substantial contributions to conception and design, analysis and interpretation of data. GK made substantial contributions to conception, design and revising the article critically for important intellectual content. WMM made substantial contributions to conception, design and revising the article for important intellectual content. The manuscript has been read and approved by all named authors or their next of kin.
